# The Extent of Necrosis in Brain Metastases May Predict Subtypes of Primary Cancer and Overall Survival in Patients Receiving Craniotomy

**DOI:** 10.3390/cancers14071694

**Published:** 2022-03-26

**Authors:** Jihwan Yoo, Yoon Jin Cha, Hun Ho Park, Mina Park, Bio Joo, Sang Hyun Suh, Sung Jun Ahn

**Affiliations:** 1 Department of Neurosurgery, Brain Tumor Center, Gangnam Severance Hospital, Yonsei University College of Medicine, Seoul 06230, Korea; jhy8486@yuhs.ac (J.Y.); nshhp@yuhs.ac (H.H.P.); 2 Department of Pathology, Gangnam Severance Hospital, Yonsei University College of Medicine, Seoul 06230, Korea; yooncha@yuhs.ac; 3 Department of Radiology, Gangnam Severance Hospital, Yonsei University College of Medicine, Seoul 06230, Korea; to.minapark@yuhs.ac (M.P.); yonnebio@yuhs.ac (B.J.); suhsh11@yuhs.ac (S.H.S.)

**Keywords:** brain metastasis, necrosis, biomarker, craniotomy

## Abstract

**Simple Summary:**

Knowledge regarding the character of necrosis in brain metastases from different primary cancer subtypes, as well as its impact on patient survival post-craniotomy, remains unknown. We performed a retrospective analysis on 145 BMs and found that lung cancers presented with a generally larger area of necrosis. Further, moderately abundant necrosis to tumor ratio appeared to confer better overall survival versus highly abundant, or even sparse necrosis. Findings of this study indicate that necrosis to tumor ratio in brain metastasis may predict subtypes of primary cancer and have potential as a biomarker for disease prognostication.

**Abstract:**

Although necrosis is common in brain metastasis (BM), its biological and clinical significances remain unknown. We evaluated necrosis extent differences by primary cancer subtype and correlated BM necrosis to overall survival post-craniotomy. We analyzed 145 BMs of patients receiving craniotomy. Necrosis to tumor ratio (NTR) was measured. Patients were divided into two groups by NTR: BMs with sparse necrosis and with abundant necrosis. Clinical features were compared. To investigate factor relevance for BM necrosis, multivariate logistic regression, random forests, and gradient boosting machine analyses were performed. Kaplan–Meier analysis and log-rank tests were performed to evaluate the effect of BM necrosis on overall survival. Lung cancer was a more common origin for BMs with abundant necrosis (42/72, 58.33%) versus sparse necrosis (23/73, 31.51%, *p* < 0.01). Primary cancer subtype and tumor volume were the most relevant factors for BM necrosis (*p* < 0.01). BMs harboring moderately abundant necrosis showed longer survival, versus sparse or highly abundant necrosis (*p* = 0.04). Lung cancer BM may carry larger necrosis than BMs from other cancers. Further, moderately abundant necrosis in BM may predict a good prognosis post-craniotomy.

## 1. Introduction

Brain metastases (BMs) occur in 10% to 20% of adult patients with cancer and are the most common malignant intracranial neoplasm, outnumbering primary malignant tumors by more than 10-fold [1,2]. Recently BMs have been recognized as an emerging area of clinical interest as progress in the development of systemic treatments, together with the rationalized use of surgical resection, radiosurgery, and whole-brain radiotherapy, have substantially prolonged survival of patients with BMs [3,4]. In particular, new targeted and immunomodulatory therapies have shown promising intracranial efficacy [5].

Despite growing interests in BMs and paradigm shifts regarding treatment, imaging features of BMs have been rarely studied. Previous studies have focused on the detection of BMs and differentiation from other malignancies, particularly glioblastoma [6,7,8,9]. However, given the current trend of personalized treatment of BMs, imaging features of BMs with different biologic subtypes are necessary.

We serendipitously noted that necrosis was a common finding in BMs and that the extent of necrosis varied according to the subtype of primary cancers, which has never been assessed. Additionally, the clinical significance of necrosis in BMs remains unknown. A common hypothesis for tumor necrosis is that the rapid growth of malignant cells outgrows the capacity of inherent blood supply, generating hypoxic conditions resulting in necrotic tissue areas [10]. However, tumor cells maintain an altered metabolic pathway known as the Warburg effect, choosing glycolysis as the primary energy source. Therefore, the degree of necrosis can vary according to tumor biological characteristics and treatment response [11,12]. A previous study reported that necrosis in breast cancer BMs was significantly associated with inferior overall survival in patients receiving craniotomy [13]. Patients with BM harboring a larger area of necrosis showed an unfavorable postoperative seizure outcome [14]. However, more evidence is necessary to draw a solid conclusion regarding the clinical impact of BM necrosis. Thus, the aims of our study were two-fold: (1) to assess if the extent of necrosis in BM was different according to the subtype of primary cancer; and (2) to determine if BM necrosis could predict overall survival in patients receiving surgical resection of BM via craniotomy.

## 2. Materials and Methods

### 2.1. Participants

This retrospective study was approved by our institutional review board, which waived the requirement for informed patient consent (3-2021-0418). We retrospectively searched through electronic medical records to identify patients who received surgical resection for brain metastases from March 2013 to December 2020. Indications for surgical resection included mass effect, controlled primary disease, high-performance score, limited number of lesions, and radio-resistance [15]. From 192 identified patients, we excluded some for the following reasons: (1) history of cranial irradiation until craniotomy (*n* = 20), (2) extra-axial location (*n* = 5), (3) non-available contrast-enhanced T1-weighted image (*n =* 4), (4) pathology other than BM (*n =* 1), and (5) small number of brain metastases for statistical analysis (liver = 9, melanoma = 4, sarcoma = 2, thyroid = 6). Finally, a total of 141 patients with 145 brain metastases (breast = 37, genitourinary = 19, gastrointestinal = 24, and lung = 65) were analyzed in this study. 137 patients received craniotomy for single metastasis. Four patients received craniotomy for two brain metastases. Patients underwent whole-brain radiation therapy (WBRT) within one month after craniotomy. WBRT was given to a total dose of 30 Gy in 10 fractions. Upon completion of WBRT, adjuvant chemotherapy was followed according to histologic, genetic, and molecular subtypes. MRI surveillance was performed at intervals of three months until 2 years after surgery, then at intervals of six months. All data were completely anonymized, and all analyses were conducted in accordance with the approved guidelines.

### 2.2. MRI

Routine magnetic resonance images (MRIs) for the evaluation of BMs were acquired using the Siemens 3T Vida (Siemens Healthineers, Erlangen, Germany) or GE 3T Discovery MR750 (GE Healthcare, Milwaukee, WI, USA) scanners. Our brain MRI protocol for the Siemens 3T scanner included T1-weighted three-dimensional (3D) magnetization-prepared rapid acquisition with gradient echo (MPRAGE) imaging (repetition time (TR)/echo time (TE)/inversion time(TI) = 2300/3/900 ms, FOV = 256 mm, voxel size = 1 × 1 × 1 mm), axial T2-weighted image (TR/TE = 5700/100 ms, FOV = 230 mm, voxel size = 0.45 × 0.45 × 5 mm), axial T2 FLAIR (TR/TE/TI = 8000/128/2370 ms, FOV = 230 mm, voxel size =0.6 × 0.6 × 5 mm), and SWI (TR/TE = 28/20 ms, FOV = 220, voxel size = 0.57 × 0.57 × 2 mm, flow compensation). A 3D turbo spin-echo T1-weighted image (SPACE) (TR/TE = 700/33 ms, FOV = 230 mm, voxel size = 0.8 × 0.8 × 0.8 mm) was additionally acquired after administering gadobutrol 0.2 mmol/kg (Gadovist, Bayer Schering Pharma; Berlin, Germany). A corresponding sequence was used with similar MR parameters for the GE scanner.

### 2.3. Image Postprocessing and Analysis

**Figure 1:**
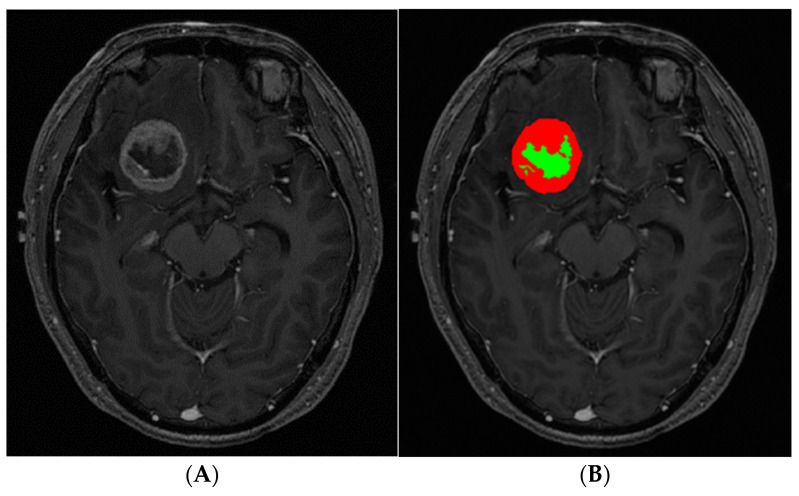
Use of a semiautomatic method to segment brain metastasis (BM) as regions of interest in a 39-year-old man with brain metastasis from lung cancer. (**A**) Contrast-enhanced T1-weighted magnetic resonance (MR) image. (**B**) Enhancing lesions (solid, red) and non-enhancing lesions (necrosis, yellow) are segmented separately.

Tumor segmentation was performed on contrast-enhanced 3D T1-weighted SPACE by using the open-source software, ITK-Snap version 3.8.0 (www.itksnap.org; accessed on 20 February 2022) [16]. Necrosis was defined as a non-enhancing area within BM based on the previous studies [17,18]. The workflow of BM segmentation was divided into three stages. In the first stage, we marked examples of different tissue classes (enhancing lesion, necrosis, and background) present in the image by using the paintbrush tool. Random forest classification produced a scalar “speed” image (g) with intensities in the range [−1,1] based on marked tissue classes. In the second stage, we initialized the active contour segmentation mode by placing one or more spherical ‘seeds’ within the earlier produced “speed” image. The evolving estimate was graphically displayed as a color label. We stopped the algorithm when there was no further algorithm growth or when the estimated tumor borders leaked outside the target lesion. These active contour segmentation processes were performed for any enhancing lesion(s) as well as for necrosis. In the third stage, the result of auto-segmentation was reviewed and revised manually by a neuro-radiologist with 8 years of experience (Figure 1). The necrosis to tumor ratio (NTR; necrosis divided by tumor volume) was calculated for further analysis. This process was independently repeated by a neuro-radiologist with 10 years of experience. The average of the NTRs from the two readers was used for further analysis.

### 2.4. Statistical Analyses

The study cohort was divided into two groups based on the threshold of the median value of NTR (0.18): BMs with sparse necrosis and BMs with abundant necrosis. Subtypes of primary cancer, sex, age, time interval from initial tumor diagnosis to BM resection, history of chemotherapy, total volume of BMs, anatomical location of BM, preoperative, Karnofsky Performance Score (KPS), and postoperative neurologic complication (hemorrhage, infection, and hydrocephalus) were compared between the two groups. Additionally, four types of primary cancers (breast, genitourinary, gastrointestinal, and lung) were further dichotomized into two groups (lung vs. other primary cancer) because lung cancer showed a significantly higher proportion of BM with abundant necrosis than other primary cancer. BM anatomical locations were also dichotomized into two groups (supratentorial vs infratentorial areas). Independent *t*-tests were used for continuous variables, while the chi-squared test or Fisher’s exact test was used for categorical variables. The inter-rater reliability was assessed using the intra-class correlation coefficient with a two-way random model of absolute agreement.

Multivariate logistic regression analysis was also performed to adjust for subtypes of primary cancer, sex, age, and tumor volume, which were statistically significant in the univariate analysis or basic clinical factors. To measure the influence of variables for predicting necrosis within BMs, variable importance scores were calculated by using two popular ensemble methods, random forests (RF) and gradient boosting machine (GBM) [1,19]. They are ensemble learning methods and predict (regression or classification) by combining the outputs from individual trees. RF refers to the process of creating and merging a collection of independent, parallel decision trees using different subsets of the training data while GBM takes an iterative approach to combine several weak, sequential models to create one strong model by focusing on the mistakes in the prior iterations.

Additionally, patients with BM from primary lung cancer were split into subgroups according to their histology, and their NTRs were compared.

To explore the predictability of BM necrosis for overall survival in patients receiving craniotomy, BMs with abundant necrosis were further divided into moderately and highly abundant necrosis with an NTR cut-off value of 0.5. Kaplan–Meier analysis was performed to evaluate the effect of NTR on patients’ overall survival. Log-rank tests were used to compare three groups (BMs with sparse necrosis, moderately abundant, and highly abundant necrosis). Additional survival analyses were also performed for non-small cell lung cancer (NSCLC) patients. To verify the independent effect of necrosis on survival, other explanatory variables (age, performance status, number of BMs, epidermal growth factor receptor (EGFR) status, and extra-cranial metastasis) were also compared among the three groups [7]. All data analyses were performed using R version 3.5.3 (The R Foundation for Statistical Computing, Vienna, Austria). A *p*-value < 0.05 was considered significant.

## 3. Results

### 3.1. Patient Characteristics

**Figure 2:**
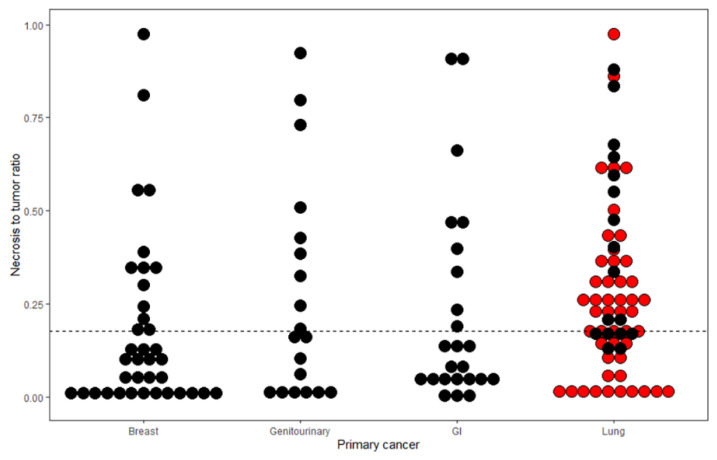
Dot plot of necrosis to tumor ratio (NTR) of BMs from different primary cancers. Adenocarcinoma in lung cancer was colored with red.

A total of 141 patients with 145 BMs were included in this study. The demographics and clinical characteristics are summarized in Table 1. Lung cancer was a more common origin for BMs with abundant necrosis (42/72, 58.33%) versus those with sparse necrosis (23/73, 31.51%, *p* < 0.01, Figure 2). BM with abundant necrosis showed a larger tumor volume than BM with sparse necrosis (25.74 ± 28.09 cm^3^ vs. 12.07 ± 15.77 cm^3^, *p* < 0.01). BM with abundant necrosis involved frontal lobe, occipital lobe, and parietal lobe more frequently but cerebellum, temporal, and subcortex less frequently than those with sparse necrosis (*p* = 0.02). No significant differences were observed regarding age, sex, time interval from initial tumor diagnosis to BM resection, history of chemotherapy, preoperative KPS, and postoperative complications. Detailed postoperative complications are described in Supplemental Table S1. Inter-observer agreement for NTR was excellent (ICC = 0.92).

### 3.2. Most Influential Variable Predicting BM with Abundant Necrosis

Multivariate analysis found that primary lung cancer was independently associated with BM with abundant necrosis (odds ratio [OR] = 3.33; 95% confidence interval [CI]: 1.49, 7.69, *p* < 0.01). Tumor volume was also independently associated (OR = 1.04; 95% CI: 1.02, 1.07, *p* < 0.01, Table 2). In ensemble methods, the variable with the highest prediction power for prediction of BM with abundant necrosis was tumor volume (mean decrease accuracy: 15.81 in RF, relative influence: 65.50 in gradient boosting) and the variable with the second highest prediction power was primary lung cancer (mean decrease accuracy: 13.47 in RF, relative influence: 23.51 in gradient boosting). The importance scores of other variables are summarized in Table 3.

### 3.3. Relationship between NTR and Lung Cancer Subtypes

**Figure 3:**
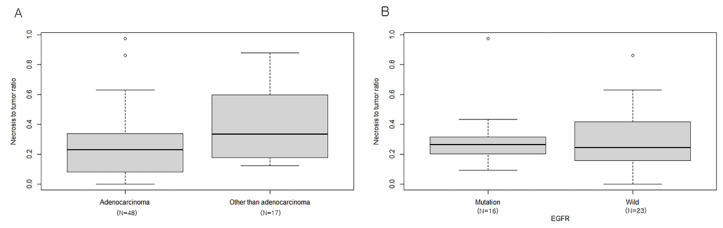
Comparison of NTR of BM from different subtypes of lung cancer. (**A**) Adenocarcinoma vs. other than adenocarcinoma (neuroendocrine carcinoma and squamous cell carcinoma). (**B**) mutant epidermal growth factor receptor (EGFR) vs. wild-type EGFR.

BMs from lung neuroendocrine carcinoma and squamous cell carcinoma showed higher NTR than those from lung adenocarcinoma (0.40 ± 0.26 vs. 0.25 ± 0.22, *p* = 0.03). NTR was not significantly different between the EGFR mutant group and wild-type group (0.30 ± 0.20 vs. 0.31 ± 0.22, *p* = 0.89, Figure 3).

### 3.4. Effect of BM Necrosis on Overall Patient Survival

**Figure 4:**
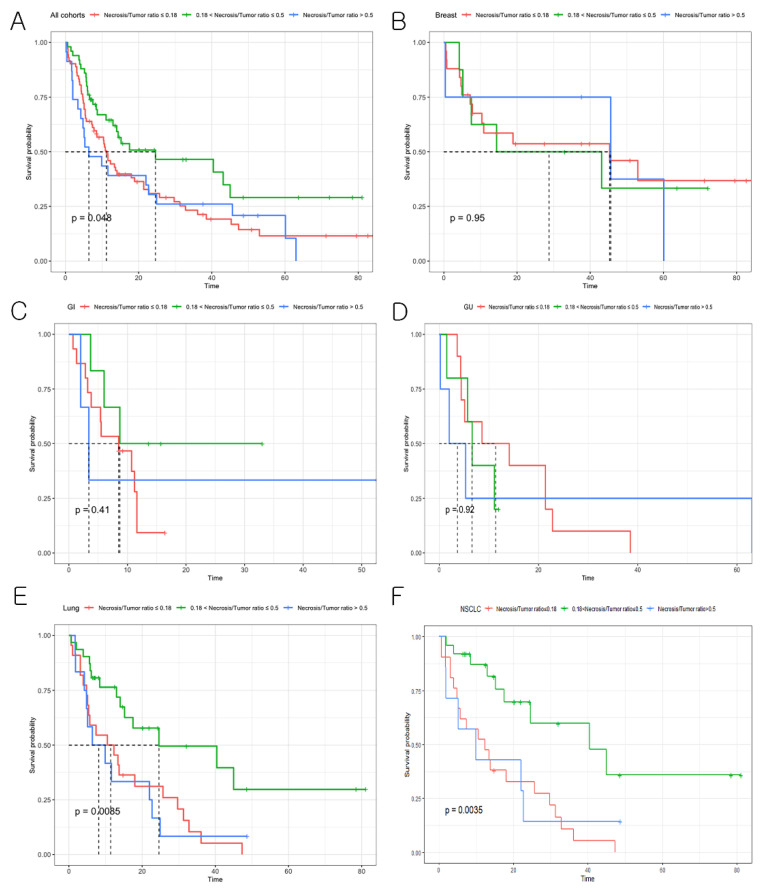
Survival analysis according to the NTR. (**A**) Kaplan–Meier survival graph for the entire cohort with BMs harboring sparse necrosis, moderately abundant necrosis, and highly abundant necrosis (*p* = 0.048). (**B**) Breast cancer. (**C**) Gastrointestinal cancer. (**D**) Genitourinary cancer. (**E**) Lung cancer. (**F**) NSCLC.

The median overall survival of patients with BMs was 13.0 months. There was a significant difference in overall survival among three groups (BMs with sparse necrosis (NTR ≤ 0.18), moderately abundant necrosis (0.18 < NTR ≤ 0.5), and highly abundant necrosis (NTR > 0.5), *p* = 0.05, Figure 4A). A post-hoc test revealed that the median overall survival for patients with BMs harboring moderately abundant necrosis was longer than that of patients with BMs harboring sparse necrosis as well as those with highly abundant necrosis (moderately abundant necrosis (median overall survival = 24.6 months) vs. sparse necrosis (median overall survival = 11.2 months), *p* = 0.02; vs. highly abundant necrosis median overall survival = 6.4 months), *p* = 0.03). 

In subgroup analysis, patients with lung cancer BMs harboring moderately abundant necrosis also showed a significantly longer overall survival than those with BMs harboring sparse necrosis as well as highly abundant necrosis (moderately abundant necrosis (median overall survival = 24.6 months) vs. sparse necrosis (median overall survival = 11.4 months), *p* < 0.01; vs. highly abundant necrosis (median overall survival = 8.2 months), *p* < 0.01, Figure 4E). However, NTR stratification did not predict overall survival in breast, gastrointestinal, and genitourinary cancer. In a subset of lung cancer, NSCLC patients with BMs harboring moderately abundant necrosis had a significantly longer overall survival than those with BMs harboring sparse necrosis as well as highly abundant necrosis (*p* < 0.01, Figure 4F). Other prognostic factors for survival of patients with BMs (age, performance status, number of BMs, EGFR status, extra-cranial metastasis, and postoperative neurologic complication) were not significantly different among the three groups (Supplemental Table S2). EGFR stratification demonstrated that the overall survival of EGFR mutant group was longer than that of EGFR wild-type group, but did not reach a statistical significance (Supplemental Figure S1).

## 4. Discussion

In this study, we tested a hypothesis that the extent of tumor necrosis in BMs may be different according to the subtype of primary cancer and that BM necrosis could be an independent predictor for overall survival in patients receiving craniotomy. Our results indicated that lung cancer BMs had more necrosis than BMs from other primary cancers. Particularly, neuroendocrine lung cancer and squamous cell lung cancer BMs demonstrated a larger NTR than adenocarcinoma lung cancer BMs. Moreover, patients with BMs harboring highly abundant necrosis had a poor prognosis, while those with BMs having moderately abundant necrosis had a good prognosis after craniotomy. Our results may elaborate on the pathophysiology of BMs and can be utilized for stratifying the overall survival of lung cancer patients with BM receiving surgical resection.

Although tumor necrosis is a common histological feature in various cancers, its underlying mechanism remains largely elusive. A common cause of tumor necrosis is metabolic stress. Tumor cells maintain a high glycolytic rate even in conditions of adequate oxygen supply [8]. However, most tumor cells are subjected to deprivation of oxygen and nutrients due to abnormal vasculature [7]. The rapidly growing tumor cells are inevitably stripped of the vascular supply, resulting in deprivation of oxygen and nutrients [10]. Moreover, recent studies suggest that immune cells may amplify tumor necrosis [20]. Necrosis is the hallmark feature of glioblastoma. Its clinical significance has been well documented. Previous studies revealed that larger necrotic areas predicted a poor prognosis [21,22]. Tumor cells surrounding necrosis that survive hypoxia are more likely to proliferate than to undergo apoptosis [23]. However, necrosis in BMs has rarely been studied.

Our results demonstrated that a larger area of necrosis was associated with a larger volume of BMs, which aligns with prior research. As a tumor grows, it is likely to be deprived of a functional vascular network, leading to it becoming more hypoxic and necrotic [24,25]. Interestingly, our study indicated that the extent of BM necrosis could be dependent on primary cancer. Specifically, BMs from the lung showed a higher portion of necrosis compared with those from other sites. This phenomenon could be cautiously explained by our subgroup analysis. Neuroendocrine carcinoma and squamous carcinoma in lung cancer tended to have a higher area of necrosis in BMs than adenocarcinoma and may have contributed to a higher necrotic feature overall in BMs from the lung. Squamous cell carcinoma is a malignant epithelial tumor that originates from the bronchial epithelium and typically shows a central, comedo-type pattern of necrosis. Meanwhile, pulmonary adenocarcinoma rarely demonstrates this alteration unless tumors are extremely large or poorly differentiated [26]. Histologic features of large cell neuroendocrine carcinoma include large cell size, areas of abundant necrosis, low nuclear/cytoplasm ratio, neuroendocrine differentiation growth pattern, and high mitotic rate. Generally, it presents with much more necrosis compared with adenocarcinoma [27]. Small cell lung cancer is also classified into neuroendocrine carcinoma and is defined as a tumor with cells that have a small size, a round-to-fusiform shape, scant cytoplasm, finely granular nuclear chromatin, and absent or inconspicuous nucleoli. Necrosis is also frequent and is often extensive [28]. In addition, recent studies have revealed that brain microenvironment is regulated by primary tumor. For example, melanoma recruit pre-existing vessels in the brain parenchyma, whereas lung cancer cells induce neoangiogenesis. Thus, tumor necrosis can be affected by a primary tumor [29,30]. Our results also demonstrated that tumor necrosis could be dependent on the anatomical location of BM. Particularly, BM with abundant necrosis was not likely to occur in the cerebellum, which is strengthened by the previous study that infratentorial BM favored higher-perfused areas, whereas supratentorial BM preferred lower-perfused ones. Thus, BM with central necrosis may occur more often in the supratentorial than infratentorial areas [31]. EGFR is a transmembrane protein with cytoplasmic kinase activity that transduces important growth factor signaling from the extracellular milieu into the cell [32]. It has been regarded as an important biomarker because patients with lung cancer BMs harboring EGFR mutations exhibit a better response to treatment as well as clinical features [33,34]. However, according to our analysis, EGFR was not associated with BM necrosis.

Since the Graded Prognostic Assessment (GPA) was generated to predict outcomes of patients who develop BMs, it has been refined with diagnosis-specific prognostic indices [35,36]. GPA prognostic factors are age, Karnofsky performance status, number of BMs, and extra-cranial metastasis. Our study suggests that BM necrosis could also be used as a predictor for outcomes in patients with BM receiving craniotomy. Previous studies reported that BM necrosis may indicate a poor prognosis. Sambade et al. analyzed 203 breast cancer patients with BM receiving craniotomy and showed that necrosis was correlated with inferior overall survival [13]. In another study of 149 patients with BMs treated with stereotactic radiotherapy, patients with tumor necrosis had a median survival of 5.4 months versus 7.2 months in patients without tumor necrosis [17]. Our results also demonstrated that the median survival of patients with BMs harboring highly abundant necrosis was shorter than that of those with BMs harboring sparse necrosis or moderately abundant necrosis. However, the novel finding in our study was that moderately abundant necrosis may imply a good prognosis for patients with BM undergoing craniotomy. These contradictory results may be explained by two aspects of tumor necrosis. Tumor necrosis is known to correlate with aggressive tumor phenotypes, while necrosis in response to chemotherapy leads to increased progression-free and overall survival in several cancer types [37,38,39]. Thus, moderately abundant necrosis in BM may reflect a response to chemotherapy, which is supported by results of NSCLC patients, wherein target agents that pass through the blood–brain barrier have recently been used [40]. However, BM necrosis may be a complicated process not only associated with response to treatment, but also with histologic feature of tumor and its interaction with microenvironment. This hypothesis should be validated in a future study with a larger population.

This study had limitations. First, certain locations of primary tumors such as liver, skin, and thyroid were excluded because of small sample size. Thus, our results may not be generalized in overall cancer. Second, our stratification of BMs was based on the median value of NTR. For a more accurate reference value, a larger population survey or meta-analysis is necessary. Third, diffusion weighted imaging (DWI) and MR spectroscopy were not included in our routine MRI protocol. Recent studies have reported that DWI metrics from BM may predict histologic type as well as patient outcomes. The association between DWI and tumor necrosis was not revealed in this study [41,42]. To prove necrosis in BM, it is necessary that MR spectroscopy shows elevated lipid and lactate [18]. However, A cystic BM showing non enhancement can be a differential diagnosis with necrotic BM but has been rarely reported [43]. 

## 5. Conclusions

Our study demonstrated that lung cancer BMs have more necrosis than BMs from other primary cancers. Particularly, neuro-endocrine lung cancer and squamous cell lung cancer BMs demonstrated a larger NTR. Moreover, the extent of necrosis in BMs can be utilized for stratifying the overall survival of lung cancer patients, with BM receiving surgical resection. BMs with moderately abundant necrosis may indicate a good prognosis.

## Figures and Tables

**Table 1:** Baseline characteristics of patients with BMs with/without abundant necrosis.

	BMs with Sparse Necrosis (*n* = 73)	BM with Abundant Necrosis (*n* = 72)	Total (*n* = 145)	*p*
Primary cancer				<0.01 *
Breast	25 (34.25%)	12 (16.67%)	37 (25.52%)	
Genitourinary	10 (13.70%)	9 (12.50%)	19 (13.10%)	
Gastrointestinal	15 (20.55%)	9 (12.50%)	24 (16.55%)	
Lung	23 (31.51%)	42 (58.33%)	65 (44.83%)	
Lung vs. others				<0.01 *
Lung	23 (31.51%)	42 (58.33%)	65 (44.83%)	
Other tumors	50 (68.49%)	30 (41.67%)	80 (55.17%)	
Sex				0.08
F	44 (60.27%)	32 (44.44%)	76 (52.41%)	
M	29 (39.73%)	40 (55.56%)	69 (47.59%)	
Age	57.62 ± 11.51	59.68 ± 12.04	58.64 ± 11.78	0.29
Time interval to BM resection (months)	33.65 ± 50.60	23.50 ± 26.16	28.61 ± 40.54	0.13
Chemotherapy				0.12
No	20 (27.78%)	29 (41.43%)	49 (34.51%)	
Yes	52 (72.22%)	41 (58.57%)	93 (65.49%)	
BM volume (cm^3^)	12.07 ± 15.77	25.74 ± 28.09	18.86 ± 23.68	<0.01 *
BM location				0.02 *
Cerebellum	27 (36.99%)	15 (20.83%)	42 (28.97%)	
Frontal	20 (27.40%)	22 (30.56%)	42 (28.97%)	
Occipital	2 (2.74%)	6 (8.33%)	8 (5.52%)	
Parietal	15 (20.55%)	26 (36.11%)	41 (28.28%)	
Temporal	5 (6.85%)	3 (4.17%)	8 (5.52%)	
Subcortex	4 (5.48%)	0 (0.0%)	4 (2.76%)	
Preoperative KPS				0.46
<70	10 (13.70%)	10 (13.89%)	20 (13.79%)	
70–80	55 (75.34%)	49 (68.06%)	104 (71.72%)	
90–100	8 (10.96%)	13 (18.06%)	21 (14.48%)	
Postoperative complication				
No	66 (90.41%)	67 (93.06%)	133 (91.72%)	0.78
Yes	7 (9.59%)	5 (6.94%)	12 (8.28%)	

F, female; M, male; BM, brain metastasis; KPS, Karnofsky Performance Score. Asterisk (*) indicates a *p*-value < 0.05.

**Table 2:** Multiple logistic regression analysis for BM with abundant necrosis.

	Odds Ratio	*p*-Value
Primary cancer		<0.01
Breast Genitourinary Gastrointestinal cancer	1.0	
Lung cancer	3.33 (1.49–7.69)	
Sex		0.81
Female	1.0	
Male	1.11 (0.47–2.54)	
Age	1 (0.97–1.03)	0.92
Tumor volume (cm^3^)	1.04 (1.02–1.07)	<0.01
BM location		0.13
Infratentorial BM	1.0	
Supratentorial BM	1.83 (0.83–4.13)	

**Table 3:** Variables of importance for the prediction of BM with abundant necrosis using random forest and gradient boosting.

	Random Forest	Gradient Boosting
	Mean Decrease Accuracy	Relative Influence
Tumor volume (cm^3^)	15.81	65.50
Lung vs. others	13.47	23.51
Sex	3.12	1.41
Chemotherapy	3.26	0.96
BM location	1.36	3.68
Time interval to BM resection (months)	−1.98	1.95
Age	0.31	2.95

## Data Availability

The data that support the findings of this study are available from the corresponding author upon reasonable request.

## Supplementary Materials

The following are available online at https://www.mdpi.com/article/10.3390/cancers14071694/s1, Figure S1: Kaplan–Meier survival graph for NSCLC patients with BMs of EGFR mutant vs. wild-type; Table S1: Surgical complications after craniotomy; Table S2: Comparison of factors comprising the graded prognostic score in the non-small cell lung cancer (NSCLC) subgroup.
