# Supplementary material for: The Extent of Necrosis in Brain Metastases May Predict Subtypes of Primary Cancer and Overall Survival in Patients Receiving Craniotomy
Source: Cancers (Basel). 2022 Mar 26;14(7):1694. doi: 10.3390/cancers14071694 (PMC8997083; doi:10.3390/cancers14071694)
Supplement: Supplementary file 1 [file cancers-14-01694-s001.zip › cancers-1626634-supplementary.pdf]

Supplementary Material: The Extent of Necrosis in Brain Metastases May Predict Subtypes of Primary Cancer and Overall Survival in Patients Receiving Craniotomy

Jihwan Yoo, Yoon Jin Cha, Hun Ho Park, Mina Park, Bio Joo, Sang Hyun Suh and Sung Jun Ahn

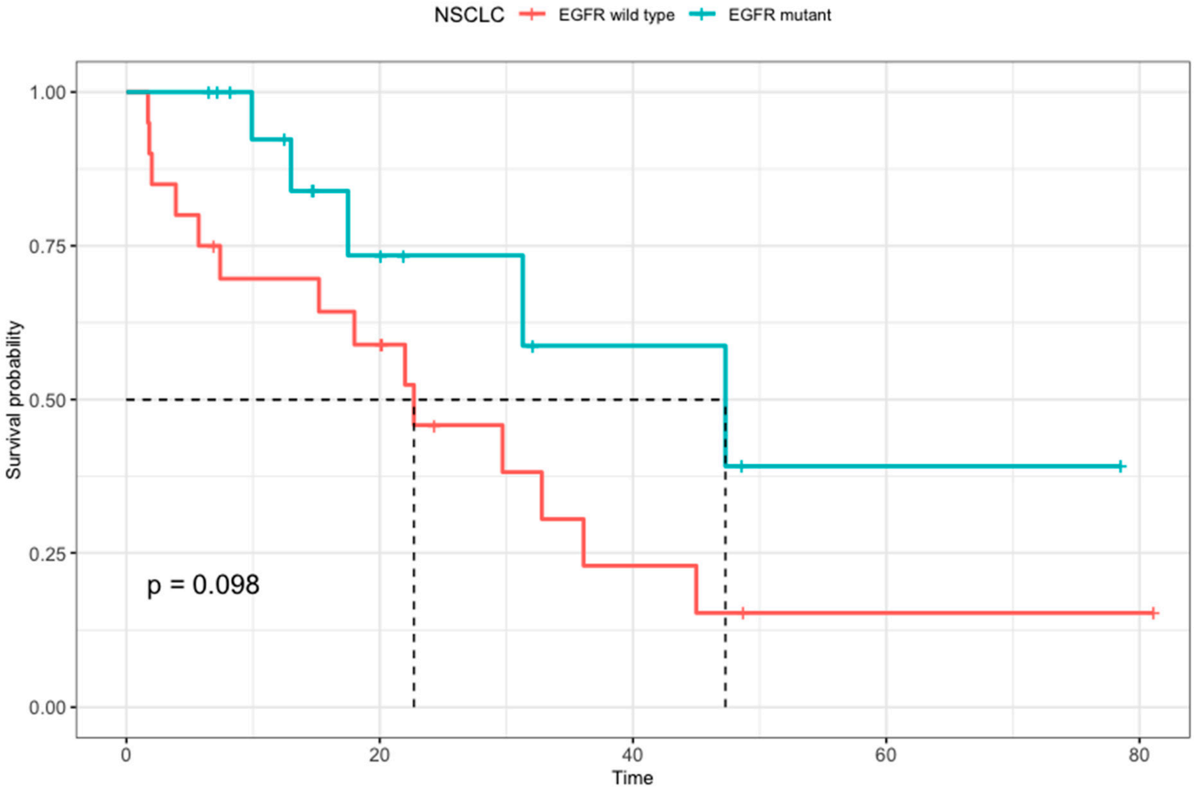

Figure S1. Kaplan–Meier survival graph for NSCLC patients with BMs of EGFR mutant vs. wild-type.

Table S1. Surgical complications after craniotomy.

**Surgical Complications**	**BMs with Sparse Necrosis**	**BMs with Abundant Necrosis**	**Total (N = 145)**	***p*-value**
	(73)	(N = 72)		
Postop hematoma				1.000
No	72 (98.6%)	72 (100.0%)	144 (99.3%)	
Yes	1 (1.4%)	0 (0.0%)	1 (0.7%)	
Infection				
No	72 (98.6%)	68 (94.4%)	140 (96.6%)	0.354
Yes	1 (1.4%)	4 (5.6%)	5 (3.4%)	
Wound dehiscence				
No	72 (98.6%)	71 (98.6%)	143 (98.6%)	1.000
Yes	1 (1.4%)	1 (1.4%)	2 (1.4%)	
Hydrocephalus				0.731

Surgical Complications	BMs with Sparse Necrosis	BMs with Abundant Necrosis	Total (N = 145)	*p*-value
	(73)	(N = 72)		
No	68 (93.2%)	69 (95.8%)	137 (94.5%)	
Yes	5 (6.8%)	3 (4.2%)	8 (5.5%)	
CSF leak				
No	72 (98.6%)	72 (100.0%)	144 (99.3%)	1.000
Yes	1 (1.4%)	0 (0.0%)	1 (0.7%)	

Table S2. Comparison of factors comprising the graded prognostic score and postoperative neurologic complication in the non-small cell lung cancer subgroup.

Prognostic Factor.	NTR ≤ 0.18 (N = 20)	0.18 < NTR ≤ 0.5 (N = 26)	NTR > 0.5 (N = 7)	*p*-value
Age, y				
≥70	4 (20.0%)	6 (23.1%)	3 (42.9%)	0.467
<70	16 (80.0%)	20 (76.9%)	4 (57.1%)	
KPS				
<70	4 (20.0%)	3 (11.5%)	0 (0.0%)	0.184
70-80	13 (65.0%)	15 (57.7%)	7 (100.0%)	
90-100	3 (15.0%)	8 (30.8%)	0 (0.0%)	
Number of BMs				
1-4	10 (50.0%)	12 (46.2%)	5 (71.4%)	0.491
>4	10 (50.0%)	14 (53.8%)	2 (28.6%)	
Extracranial metastasis				
Absent	12 (60.0%)	14 (53.8%)	4 (57.1%)	0.916
Present	8 (40.0%)	12 (46.2%)	3 (42.9%)	
Gene status				
EGFR neg and ALK neg	11 (55.0%)	9 (34.6%)	3 (42.9%)	0.574
EGFR pos or ALK pos	4 (20.0%)	9 (34.6%)	3 (42.9%)	
NA	5 (25.0%)	8 (30.8%)	1 (14.3%)	
GPA				
0-1.0	3 (15.0%)	4 (15.4%)	2 (28.6%)	0.875
1.5-2.0	7 (35.0%)	7 (26.9%)	3 (42.9%)	
2.5-3.0	7 (35.0%)	9 (34.6%)	1 (14.3%)	
3.5-4.0	3 (15.0%)	6 (23.1%)	1 (14.3%)	
Postoperative neurologic complication				
Absent	19 (95.0%)	24 (91.7%)	6 (85.71%)	0.706
Present	1 (5.0%)	2 (8.3%)	1 (14.29%)	

NTR, necrosis to tumor ratio; KPS, Karnofsky performance status; BM, brain metastasis; EGFR, epidermal growth factor receptor; ALK, anaplastic lymphoma kinase; pos, positive; neg, negative; NA, not available; GPA, Graded Prognostic Assessment
